# Second Primary Malignancy in Ixazomib Maintenance Therapy for Multiple Myeloma: Case Report and Literature Review

**DOI:** 10.1155/crh/6814237

**Published:** 2025-11-18

**Authors:** Ying Wang, Chun-Li Xu, Dong-Ping Huang, Yu Chen

**Affiliations:** ^1^Department of Hematology, The Second Affiliated Hospital of Wannan Medical College, Wuhu, Anhui, China; ^2^The People's Hospital of Dongtai City Affiliated to Jiangsu Vocational College of Medicine, Dongtai, Jiangsu, China; ^3^Department of Laboratory Medicine, The People's Hospital of Dongtai City, Dongtai, Jiangsu, China; ^4^Department of Hematology, The First Affiliated Hospital of Wannan Medical College, Wuhu, Anhui, China

**Keywords:** ixazomib, multiple myeloma, myelodysplastic syndrome

## Abstract

**Objective:**

With the prolongation of life expectancy among multiple myeloma (MM) patients, the development of second primary malignancies (SPMs) has emerged as a serious issue, so it is worthwhile to explore the mechanisms and therapeutic strategy regarding SPMs secondary to MM.

**Case report:**

We describe a patient with MM who developed secondary myelodysplastic syndrome (MDS) after 5 years of maintenance chemotherapy with ixazomib.

**Discussion:**

In our case, the patient was young and did not have a cytogenetic examination; after a maintenance therapy with ixazomib for about 5 years, he developed the MDS. He was subsequently recommended for allogeneic hematopoietic stem cell transplantation (allo-HSCT) and remains alive.

**Conclusions:**

The possibility of an association between ixazomib maintenance treatment and increased SPMs cannot be excluded, requiring future studies with large samples.

## 1. Introduction

Over the past two decades, the introduction of new treatment modalities—such as immunomodulatory drugs (IMiDs, including thalidomide and lenalidomide), proteasome inhibitors (bortezomib, carfilzomib, and ixazomib), and anti-CD38 monoclonal antibodies (e.g., daratumumab)—has significantly improved progression-free survival (PFS) and overall survival (OS) in patients with multiple myeloma (MM). With OS in this patient population improving, therapy-related neoplasms or second primary malignancies (SPMs) become more evident [1]. Ixazomib [2], a second-generation proteasome inhibitor, is emerging as a central therapeutic option for the treatment of MM. However, there are few reports of SPMs secondary to the treatment with ixazomib [3]. Here, we report a patient with MM who developed secondary myelodysplastic syndrome (MDS) after 5 years of maintenance chemotherapy with ixazomib.

## 2. Case Report

**Figure 1:**
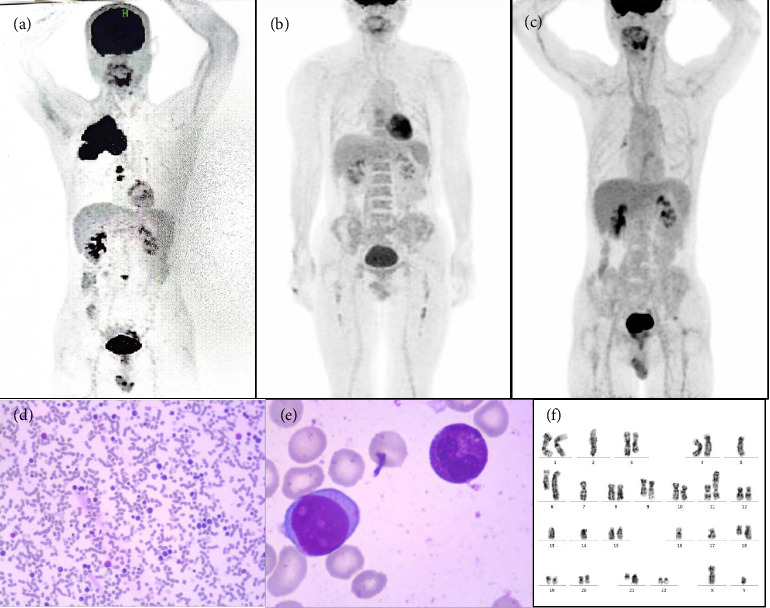
The laboratory examination of the patient. (a) 2018.07 PET-CT showed hypermetabolic in the right anterior superior mediastinum and right thoracic chest wall (SUVmax = 14). (b) 2019.08 PET-CT showed no obvious hypermetabolic foci. (c) 2024.11 PET-CT showed no obvious hypermetabolic foci. (d) Bone marrow aspirate (Wright–Giemsa stain, 4×) showing markedly hypercellular marrow with clusters of immature myeloid precursors. (e) High-power field (100×) of bone marrow aspirate revealing approximately 12% primitive myeloid blasts characterized by a high nuclear-to-cytoplasmic ratio, fine chromatin, and prominent nucleoli. (f) Chromosome analysis revealed a complex karyotype of 39∼40, XY, −2, add(3)(q12), −5, add(6)(q23), −7, add(9)(q34), add(11)(p15), add(12)(p12), −13, −14, −16, −17, add(18)(q23), −21, add(21)(q22), inc[cp15]/46, XY [5].

**Figure 2:**
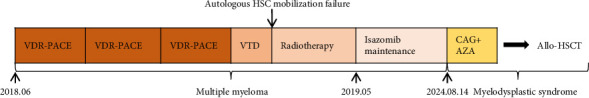
The treatment regimen of the patient.

A 54-year-old male presented to the hospital in June, 2018, with complaints of chest tightness. Enhanced CT of the chest suggested an 8.8 × 2.8 cm mass on the right anterior upper wall chest, with moderate enhancement, invading the chest wall and the right second anterior rib. Pathology of the mass was positive for CD38, MUM1, CD79a, and Lamda. Bone marrow (BM) examination showed 1% plasma cells. The immunophenotype indicated 0.3% blasts with plasma cell markers, which were positive for CD45, CD38, CD138, and cLambda and negative for kappa. BM biopsy showed increased plasma cells. Positron emission tomography-computed tomography (PET-CT) showed hypermetabolic in the right anterior superior mediastinum and right thoracic chest wall (SUVmax = 14) (Figure 1(a)). He was diagnosed with MM and underwent VDR-PACE for 3 cycles and VTD for 1 cycle. Autologous hematopoietic stem cell transplantation was not performed due to failed mobilization, and 20 times radiotherapy for the mass was performed. Then the patient started the ixazomib maintenance therapy in May, 2019, with regular monitoring (Figures 1(b) and 1(c)). On August 14th, 2024, a complete blood count (CBC) revealed the following: WBC 0.94 × 10^9^/L, Hb 95 g/L, and PLT 34 × 10^9^/L. BM examination (Figures 1(d), 1(e), and 1(f)) revealed the presence of 12% primitive myeloid cells, 19% ringed ferritic erythrocytes, and 0.5% plasma cells. The immunophenotype indicated 7.5% blasts with myeloid cell markers, which were positive for HLA-DR, CD13, CD33, CD34, CD38, CD58, CD117, and CD123. Next-generation sequence (NGS) indicated that TET2 (c.2884C > T p.Q962Ter VAF: 37.98%) and TP53 (c.273G > A p.W91Ter VAF: 40.28%) mutations were positive. Chromosome analysis (Figure 1(f)) revealed a complex karyotype of 39–40, XY with multiple numerical and structural abnormalities, including −2, add(3)(q12), −5, add(6)(q23), −7, add(9)(q34), add(11)(p15), add(12)(p12), −13, −14, −16, −17, add(18)(q23), −21, and add(21)(q22), observed in a composite of 15 metaphases (inc[cp15]). In addition, a normal male karyotype, 46, XY, was detected in 5 metaphases [5]. Then the patient was diagnosed with secondary MDS. He received the azacitidine (AZA) combined with CAG chemotherapy and got complete remission (CR). Since this patient had no sibling donor, an unrelated donor allogeneic hematopoietic stem cell transplant (allo-HSCT) was recommended and he remains alive. The treatment regimen is illustrated in Figure 2.

## 3. Discussion

With the continuous improvement in the survival of MM patients, SPMs have become an increasingly relevant long-term risk for MM survivors [4]. Since the 1950s, population studies have consistently observed an increased incidence of hematological SPMs in MM survivors.

Recently, some researchers have suggested that cancer can be viewed as a pathological ecosystem rather than only a genetic disease. This ecological and evolutionary view highlights how tumor cells and their microenvironment interact and adapt under treatment pressure [5]. Gatenby proposed “adaptive therapy,” which aims to control tumor burden over time instead of complete eradication by maintaining a balance between sensitive and resistant cells [6]. Although MM is a hematological malignancy, similar evolutionary pressures may contribute to the development of SPMs. This perspective may help to explain their occurrence and provide new ideas for future prevention and treatment. The pathogenesis of SPMs in MM is likely to be multifactorial, depending on patient-, disease-, and treatment-related factors. It is possible that advanced age may be an adverse risk factor for the development of SPMs, given that age-related clonal hematopoiesis has been associated with an increased risk of hematological cancers [7]. Additionally, heterogeneity in MM cytogenetics has been linked to the emergence of SPMs. A single-center retrospective study reported that 77% of MM patients who developed SPMs exhibited complex/high-risk cytogenetics [8]. With regard to treatment-related factors, prolonged treatment with alkylating agents, particularly melphalan [9], autologous stem cell transplantation [10], and IMiDs, especially lenalidomide [11], has been linked to an elevated risk of hematological SPMs. It has been reported that the time intervals between radiation-induced SPMs typically vary from 8 to 21 years [12]. Newer anti-myeloma therapies including proteasome inhibitors and monoclonal antibodies did not appear to increase the risk of SPM, although the lack of robust long-term follow-up limits the strength of this conclusion. In our case, the patient was young and lacked cytogenetic examination data at diagnosis. After roughly 5 years of ixazomib maintenance therapy, MDS occurred. This observation suggests that an association between proteasome inhibitors like ixazomib and an elevated risk of SPMs may exist, though this requires further verification. It has been reported that the median survival of patients with MM with SPMs was (35.29 (19.66–50.91) months), which is significantly shorter than that of patients without SPMs (51.05 (46.7–55.4) months) [6, 13]. A retrospective study on behalf of the Chronic Malignancies Working Party of the EBMT revealed that survival of SPMs will benefit from allo-HSCT [14]. The patient in our case was advised to undergo allo-HSCT and remains alive as of the latest follow-up. In the future, it is worthwhile to investigate the mechanisms and therapeutic strategy regarding SPMs secondary to MM.

## Data Availability

The data supporting this study's findings are available from the corresponding author upon reasonable request.
